# Monobromated Camphor

**Published:** 1889-07

**Authors:** 


MONOBROMATED CAMPHOR.—Seeing a prescription of monobromated camphor for dysentery in the last number of our Journal, a medical friend writes:

"Will you be so kind as to write me the sphere of effectiveness and usefulness you attribute to monobromated camphor? To which we answer that we have rarely used this preparation except in the summer diarrhœa and dysenteries of infants, especially of teething children. Bromated camphor, says Wood, was first introduced by Prof. Deneffe (Pres. Med. Bilge, 1871) as a nervous sedative, and as an antispasmodic. Our present knowledge of the physiological properties are derived from experiments upon frogs by Bourneville and of Pellicani in 1874, in which it was shown that the drug *impairs reflex excitability*. It is this reflex excitability in teething children that brings about the convulsions and brain troubles so fatal in that class of sufferers.

The best form for using the remedy in infants is the following:

| | | |
|---|---|---|
| ℞ | Monobromated camphor, | grs. x. |
| | Sugar of milk, | grs. 90. |

M. and triturate thoroughly. Dose, 1 to 4 grs every two, three or four hours, according to the urgency of the case. As the discharges become less frequent it will suffice to give one after each evacuation until sufficiently controlled.

The remedy restrains the bowels, and promotes sleep, but does not endanger the brain or disturb the secretions like opium, and may therefore be substituted for paregoric in many instances with improved results.

To adults we sometimes combine it, in larger doses, with morphine or Dover's powder, in dysentery, after the action of calomel, and with happy effect.

